# A 3-year prospective study of the effects of adjuvant treatments on cognition in women with early stage breast cancer

**DOI:** 10.1038/sj.bjc.6603029

**Published:** 2006-03-07

**Authors:** V Jenkins, V Shilling, G Deutsch, D Bloomfield, R Morris, S Allan, H Bishop, N Hodson, S Mitra, G Sadler, E Shah, R Stein, S Whitehead, J Winstanley

**Affiliations:** 1 Cancer Research UK Psychosocial Oncology Group, Brighton and Sussex Medical School, University of Sussex, East Sussex BN1 9QG, UK; 2 Sussex Cancer Centre, Royal Sussex County Hospital, Eastern Road, Brighton, East Sussex BN2 5BE, UK; 3 Eastbourne District Hospital, Kings Drive, Eastbourne, East Sussex BN21 2UD, UK; 4 Royal Bolton Hospital, Minerva Road, Farnworth, Bolton BL4 0JR, UK; 5 Conquest Hospital, The Ridge, St Leonards-on-Seax, East Sussex TN37 7RD, UK; 6 Ludwig Institute for Cancer Research, Courtauld Building, 91 Riding House Street, London W1W 7BS, UK

**Keywords:** breast cancer, chemotherapy, endocrine treatment, cognitive dysfunction

## Abstract

The neuropsychological performance of 85 women with early stage breast cancer scheduled for chemotherapy, 43 women scheduled for endocrine therapy and/or radiotherapy and 49 healthy control subjects was assessed at baseline (T1), postchemotherapy (or 6 months) (T2) and at 18 months (T3). Repeated measures analysis found no significant interactions or main effect of group after controlling for age and intelligence. Using a calculation to examine performance at an individual level, reliable decline on multiple tasks was seen in 20% of chemotherapy patients, 26% of nonchemotherapy patients and 18% of controls at T2 (18%, 14 and 11%, respectively, at T3). Patients who had experienced a treatment-induced menopause were more likely to show reliable decline on multiple measures at T2 (OR=2.6, 95% confidence interval (CI) 0.823–8.266 *P*=0.086). Psychological distress, quality of life measures and self-reported cognitive failures did not impact on objective tests of cognitive function, but were significantly associated with each other. The results show that a few women experienced objective measurable change in their concentration and memory following standard adjuvant therapy, but the majority were either unaffected or even improve over time.

There are various chemotherapy regimens in use to treat early stage breast cancer, some more toxic than others, yet two women receiving the same combination of drugs may feel completely different during the course of their treatment. Whereas a number of patients may experience few side effects and are able to continue with their usual activities, others’ lives are significantly disrupted. One of the growing concerns among patients, clinicians, neuropsychologists and other health professionals is that adjuvant treatments for breast cancer may affect cognition.

It is important to recognise that the ‘cognitive dysfunction’ associated with chemotherapy is not as severe as that found with acute amnesia or presenile dementia. Rather it is more usual for women to complain of feelings of ‘fuzzy headedness’ or ‘mental slowness’, sometimes described as ‘chemo-fog’ (Schagen *et al*, 2002). Evidence from the existing literature suggests that around 16–75% of breast cancer patients receiving high and standard dose chemotherapy experience some degree of cognitive dysfunction (Wieneke and Dienst, 1995; Tchen *et al*, 2003). Hypotheses to explain these changes include, chemotherapy having a direct toxic effect on the brain, changes in circulating hormones particularly in those women who experience a treatment-induced menopause following chemotherapy, plus fatigue, anxiety and depression (McAllister *et al*, 2004).

Objective cognitive impairment is not apparent in all women, yet subjectively many women report changes in their memory and attention (Shilling *et al*, 2005a). Much of the earlier published objective data were derived from cross-sectional studies, with only two containing pretreatment (baseline) assessments (Wefel *et al*, 2004; Bender *et al*, 2005). In the former, a third of patients were reported to have cognitive impairment at baseline in one or more domains and this figure increased to 61% postchemotherapy, with 50% improving by the 12-month assessment. In the latter study, women who received chemotherapy showed impairment on a verbal memory measure, whereas those who had received chemotherapy plus tamoxifen declined on visual, verbal and working memory. Most of these differences were detected by the 18-month assessment. Both studies are limited, however, by their small sample size (*n*=18 and *n*=22 at completion of study, respectively), making meaningful conclusions difficult. A more recent preliminary analysis of 50 chemotherapy patients with 43 healthy controls showed that at the postchemotherapy assessment, 34% of patients compared with 19% of healthy control subjects experienced reliable cognitive decline relative to baseline performance on multiple measures (Shilling *et al*, 2005a). Of interest was that eight out of 17 (47%) of these patients experienced a treatment-induced menopause following chemotherapy.

The potential effect of endocrine therapy on cognitive function is more difficult to assess because of confounding factors. Women with breast cancer who receive only endocrine treatment tend to be postmenopausal and therefore older than those who receive chemotherapy. One cross-sectional study of cognitive function in women who participated in the Arimidex and Tamoxifen, Alone or in Combination (ATAC) trial noted that verbal memory and processing speed were impaired compared to healthy postmenopausal women (Jenkins *et al*, 2004). Another study reported that tamoxifen users consulted their physician about memory problems more often than nonusers (Paganini-Hill and Clark, 2000) , but as yet no study has reported longitudinal data on the effects of either tamoxifen or aromatase inhibitors on memory and attention.

Establishing the prevalence, clinical significance and impact on quality of life of cognitive impairment following breast cancer treatments is important. At the moment, the literature is too confusing to permit clear discussion. There is also some concern that the problems have been overestimated, particularly in the light of a recent meta-analysis (Falleti *et al*, 2005), which suggests that although statistically significant, the magnitude of effect sizes previously reported is small to moderate ranging from −0.03 to −0.51 of a standard deviation below controls. This report presents results from a prospective longitudinal study of the impact of breast cancer treatments on the cognitive functioning of 128 women at three time points.

## MATERIALS AND METHODS

### Participants

Women with early breast cancer from hospitals across the UK were invited to join the study following surgery but before the start of adjuvant therapy. Exclusion criteria included advanced disease, previous treatment for *any* cancer, receiving neo-adjuvant chemotherapy, those with a previous history of stroke dementia, degenerative disease and alcohol or drug abuse. Of 224, 153 (68%) patients were recruited to the study; of the 69 who did not, 39 were not interested, 11 were not eligible owing to age (>75 years) or previous treatment history and 19 did not complete the baseline assessment before the start of treatment. The control group was a sample of convenience made up of friends and family of the patients and experimenters and from a local women's group.

Of 153, 100 women were scheduled to receive adjuvant chemotherapy and 53 to receive radiotherapy and/or endocrine therapy or no further treatment. Data are presented on 128 breast cancer patients (85 received chemotherapy, 43 did not) and 49 healthy controls. Of the 15 chemotherapy patients (five no longer wanted to take part, four could not be contacted, one owing to ill-health (unrelated), one owing to disease progression and four had died of their disease); 10 nonchemotherapy patients (four no longer wanted to take part, one was not contactable, two withdrew for unrelated health reasons, two because of disease progression and one had died of causes unknown to the authors) and three healthy controls (one for health reasons, one for family reasons and one emigrated) did not complete all assessments. A further six healthy controls were excluded prior to analyses in order to bring the groups into closer alignment for age and full-scale intelligence. The study had local ethics committee approvals and all participants gave full written consent.

Table 1 shows the characteristics for the three participant groups. The nonchemotherapy group differed significantly from the healthy controls and chemotherapy group by age (*F*=22.74, *P*<0.0001; *F*=20.09, *P*<0.0001, respectively) and from the healthy control group by years spent in full time education (*F*=5.58, *P*=0.02). These differences are accounted for in the analyses. The chemotherapy group were less likely than the other groups to be peri- or postmenopausal at baseline (*χ*^2^=4.90, *P*=0.027; *χ*^2^=10.87, *P*=0.001 for healthy controls and nonchemotherapy, respectively) and the nonchemotherapy and control groups were not more likely to have used HRT.

Table 2 details the tumour grade and type of surgery for both patient groups and chemotherapy regimen. In total, 59% of the chemotherapy and 14% of the nonchemotherapy patients were lymph node positive. Time between surgery and baseline assessment ranged from 21 to 83 days (mean 41.29 s.d. 13.30) in the chemotherapy group (date of surgery missing for six patients) and 22 to 92 days in the nonchemotherapy group (mean 53.21s.d. 16.03). Eighty eight percent (75 out of 85) of the chemotherapy and 74% (32 out of 43) of the nonchemotherapy patients were seen within 2 weeks of their second assessment and at T3, the proportions were 91% (77 out of 85) and 86% (37 out of 43), respectively.

At T1, 40 out of 43 (93%) nonchemotherapy patients had started endocrine therapy; 36 received tamoxifen and four anastrozole (one went on to have goserelin injections, six switched endocrine treatment during the study and one ceased endocrine treatment) and between T1 and T2 assessments, 36 out of 43 (84%) had completed a course of radiotherapy. By T2, 20 out of 85 (23%) chemotherapy patients had started endocrine therapy; 17 received tamoxifen, three letrozole and by T3, this had risen to 60 (71%); 46 tamoxifen, nine letrozole and five anastrozole (two patients switched from tamoxifen to anastrozole between assessments T2 and T3, and two received trastuzumab). Of the patients, 16 (19%) had started a course of radiotherapy by T2 and by T3, 71 (83.5%) had completed radiotherapy treatment. Out of 39, 32 premenopausal chemotherapy patients experienced a treatment-induced menopause.

### Assessments

Cognitive assessments were made at baseline (T1), 4 weeks after the final chemotherapy session (six months in the other groups) (T2), and 12 months after the final chemotherapy session (18 months in the other groups) (T3).

**Figure 1:**
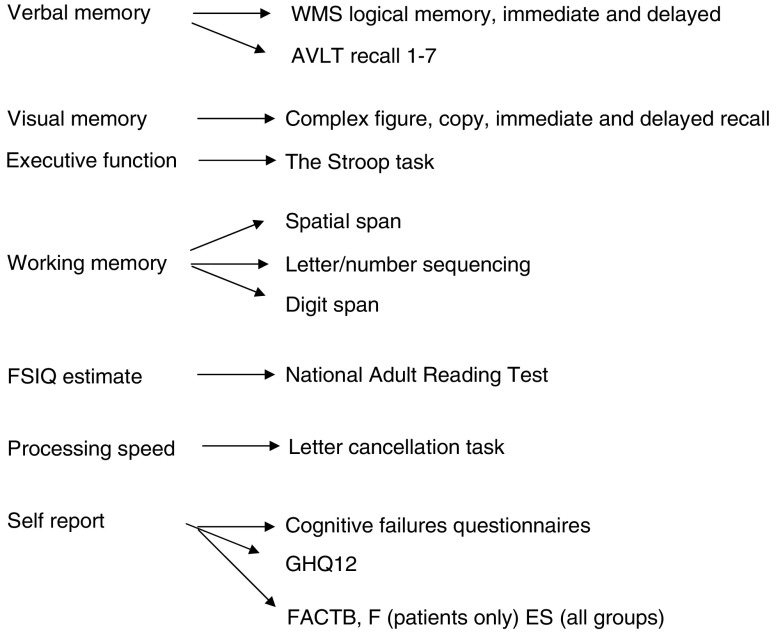
Cognitive test battery.

The cognitive test battery assesses several broad areas of cognitive function as outlined in Figure 1. The tasks used are sensitive and have shown changes in a number of patient groups, including those suffering with AIDS, Parkinson's disease, head injury, and following cardiac surgery. They can provide information on a variety of cognitive processes including attention, learning, memory, planning and organisational strategies.

The tests were administered in the same order following the requirements of the Wechsler Memory Scale-III (Weschler, 1998). All participants were screened for dementia, using the information and orientation subtest of the WMS III. The battery of standardised neuropsychological tests is briefly described below.

### Intelligence

Intelligence was assessed using the National Adult Reading Test (Nelson, 1991). Wechsler Adult Intelligence Scale FSIQ was predicted from this score.

### Verbal memory

WMS III logical memory part 1 and 2 indicate: immediate and delayed recall of a short paragraph, respectively. Rey Auditory-verbal learning test (Rey, 1964) is a word list-learning task consisting of five verbal presentations with recall of a 15-word list. Three scores are reported from this test: supraspan score (number of words recalled from the first presentation of the list), total recall score (total words recalled from the first five presentations) and delayed recall score (total words recalled after half an hour delay).

### Visual memory

Complex figure task with two alternate forms (Rey, 1941) (Taylor, 1979): copy, immediate and delayed recall of a complex geometric figure.

### Working memory

WMS III letter-number sequencing: sequences of letters and numbers must be reordered giving numbers first in ascending order and then letters in alphabetical order. WMS III digit span: strings of digits must be repeated in the same and then in the reverse order to presentation. WMS III spatial span: spatial patterns must be reproduced first in the same and then in the reverse order to presentation.

### Executive function

The Stroop task (Golden, 1978) has three conditions, colour word reading, colour patch naming and the interference condition in which colour words are printed in incompatible ink colour. The participant names the colour of the ink, requiring the inhibition of the more salient word reading.

### Processing speed and vigilance

Processing speed and vigilance are assessed using a letter cancellation task. A composite score is calculated based on both speed and accuracy.

### Self-report measures

All participants completed the General Health Questionnaire 12 (GHQ_12_) and the Broadbent cognitive failures questionnaire (Broadbent *et al*, 1982). The GHQ_12_ is a 12-item general health measure designed to screen for probable, nonpsychotic psychiatric disorder in community and medical settings (Goldberg and Williams, 1988). The cognitive failures questionnaire comprises a series of 25 questions relating to lapses in attention in everyday life, such as forgetting what the person went into a room to do. Questions are rated on a five-point scale ranging from 0-‘never’ to 5-‘very often’. As part of a structured interview (data to be presented elsewhere), patients were also asked at T2 and T3 whether they had noticed any changes in their memory and attention. Patients completed the Functional Assessment of Cancer Therapy questionnaire (Breast) (FACT B) (Brady *et al*, 1997) and the fatigue (F) subscale (Yellen *et al*, 1997). In addition, all participants completed a quality of life measure of endocrine symptoms (ES) (Fallowfield *et al*, 1999).

### Statistical methods

The Statistical Package for the Social Sciences (SPSS) version 11.5 was used for all statistical analyses. Baseline cognitive performance on each measure was analysed using stepwise multiple regression with the predictor variables of treatment group, age, FSIQ, education, psychological distress and menopausal status. Group comparisons on cognitive performance on each measure and on self-reported cognitive failures were made at the three time points, using repeated measures ANOVA with group as the between-subjects factor and time point as the within-subject factor. Where significant main effects of group were found, any variable that had significantly predicted baseline performance on that task was covaried.

Such group comparisons do not identify impairment in subgroups of the population or account for practice effects. To examine performance at an individual level, we used the reliable change index (RCI) with a correction for observed practice effects on each measure (Sawrie *et al*, 1996). By using the method put forward by Jacobson and Truax (1991), an RCI was calculated for each cognitive measure using the baseline and T2 and baseline and T3 data of the control subjects (see Appendix 1).

## RESULTS

The result section is divided into changes in performance within and between the groups over time, followed by the proportion of individuals whose performance either improved or declined over time for each task, compared with their baseline performance. The results from these objective cognitive tasks are then compared with the patients’ subjective measures of changes in psychological distress, memory and concentration and quality of life.

### Group comparisons

#### Baseline (T1)

Combinations of education, age and FSIQ consistently predicted cognitive performance (see Table 3). Treatment group failed to significantly predict cognitive performance on any of the 14 measures independently of factors such as age, education and FSIQ, as did menopausal status and GHQ_12_ scores. In all cases, the regression was a poor fit describing between 8 and 22% of the variance (*R*^2^adj between 8 and 20%), but the overall relationship was significant (*P*<0.0001) in all cases.

#### Repeated measures ANOVA

Repeated measures ANOVAs with group (chemo *vs* nonchemo *vs* control) as the between-subject factor and time point (T1 *vs* T2 *vs* T3) as the within-subject factor were conducted for each of the 14 cognitive measures. In a number of cases, the assumption of sphericity was violated. In all cases *ϵ* was greater than 0.75, so the Huynh and Feldt correction was used. Bonferroni was used for *post hoc* comparisons where sphericity was violated; Games-Howell was used where sphericity was not violated.

None of the measures showed a significant group × time point interaction. Six measures showed a significant main effect of time, with performance increasing linearly (letter cancellation *F*=15.57 *P*<0.0001; AVLT supraspan *F*=4.083, *P*=0.018; AVLT total *F*=3.175, *P*=0.043; complex figure immediate recall *F*=6.99=0.002; complex figure delayed recall *F*=10.69, *P*<0.0001; digit span backwards *F*=3.53, *P*=0.031 and Stroop *F*=4.29, *P*=0.014).

Five measures showed a significant main effect of group: AVLT supraspan (*F*=5.60, *P*=0.004); complex figure immediate recall (*F*=3.79 *P*=0.024); complex figure delayed recall (*F*=3.55, *P*=0.031); spatial span forwards (*F*=3.72, *P*=0.026) and spatial span backwards (*F*=3.71, *P*=0.026). *Post hoc* comparisons revealed significant differences were between the nonchemotherapy patient group and the healthy control group (*P*=0.004; 0.021; 0.026; 0.039; 0.036 respectively). These five repeated measures ANOVAs were recalculated covarying factors that were significant predictors of performance at baseline (age, FSIQ and in the case of spatial span backwards this also included years in education). Group ceased to be a significant main effect after covarying these baseline predictor variables, although a trend towards an effect of group remained for AVLT supraspan (*F*=2.68, *P*=0.071).

#### Reliable change analyses

The proportion of each group showing reliable decline or reliable improvement for each task is shown for both time points in Table 4. Group did not significantly predict reliable decline on any individual measure at either time with the exception of spatial span forward at T2, which was more common in the healthy control group than in either patient group (overall *χ*^2^=13.44, *P*=0.001). Those who showed reliable decline were not significantly older, or less intelligent; nor were they more likely to have above threshold GHQ_12_ scores. Reliable improvement on two or more measures was not more common in any one group at T2, but at T3 multiple improvement was more likely in the patient groups, OR 2.78 (*χ*^2^=4.925, *P*=0.02) and 2.54 (*χ*^2^, n.s.) for chemotherapy and nonchemotherapy, respectively Table 5.

#### The effect of endocrine therapy

It was not possible to compare the effects of endocrine therapy on cognitive performance within the nonchemotherapy group as 91% were receiving endocrine therapy. Within the chemotherapy group, performance did not differ significantly on any measure between those who did or did not receive endocrine therapy at T3 (*N*=60 and 25, respectively).

#### Psychological distress (GHQ_12_)

Table 1 shows the proportion of each group with above threshold GHQ_12_ scores, suggesting probable psychiatric morbidity at each time point. In both patient groups, rates of psychological distress were above 50% at baseline. In the nonchemotherapy group, this dropped significantly by T2 to levels approximately the same as the control group, where it remained at T3. In the chemotherapy group psychological distress remained high at T2 and did not drop to control levels until T3.

#### Self-reported cognitive failures

Patients and controls reported similar numbers of cognitive failures in everyday life on the Broadbent Cognitive Failures questionnaire. Repeated measures ANOVA with time point as the within-subject factor and group as the between-subject factor found no significant main effect of group but a significant main effect of time point (*F*=16.02, *P*<0.0001) and a significant interaction (*F*=4.26, *P*=0.016). The scores show a different pattern for the three groups. In the healthy control group, scores were stable across the three time points (mean score 40.14, 40.41 and 40.08). In the chemotherapy group mean, scores were initially lower than in the healthy control group (37.95), rising significantly at T2 to 43.13 (*t*=−4.24, *P*<0.0001) before dropping to 41.56. Finally, the nonchemotherapy group also had mean scores lower than the healthy control group at baseline (37.64) but showed a steady increase over time with an overall significant increase (*F*=13.34, *P*=0.001) but no significant increase between consecutive time points.

At each time point, participants with above threshold GHQ_12_ scores reported significantly more cognitive failures than those with below threshold scores (*t*=3.397, *P*=0.001; *t*=3.81, *P*<0.0001; *t*=3.22, *P*=0.002), but self-reported cognitive failures did not significantly correlate with objective cognitive test scores. At interview, the majority of the chemotherapy group reported that they had noticed changes in their memory (83%) and concentration (80%) at T2. This fell to 60 and 45%, respectively, by T3. The incidence was lower in the nonchemotherapy group with 45 and 38%, respectively, noticing changes in memory and concentration at T2. However, at T3 the proportion reporting memory problems had risen to 59%, although concentration problems remained lower at 37%. Reporting of problems was significantly associated with GHQ_12_ threshold scores but not with objective measures of cognition.

#### Quality of life and cognitive function

There was no significant main effect between the two patient groups on the fatigue subscale; however, there was a significant interaction (*F*=3.91, *P*=0.021) and a significant main effect of time (*F*=5.99, *P*=0.03). Although fatigue levels in the nonchemotherapy group were stable at all three times, the chemotherapy group showed a significant increase in symptoms immediately after chemotherapy (*t*=3.66, *P*<0.0001), with a significant improvement from baseline 1 year later (*t*=4.75, *P*<0.0001). A similar pattern was seen on the FACT-B scores (*F*=10.24, *P*=0.0001, *F*=3.43, *P*=0.034 for time and interaction, respectively). Scores in the nonchemotherapy group were generally higher and stable at the three times, whereas the chemotherapy group showed a significant improvement in quality of life at T3 (*t*=5.70, *P*<0.0001). Scores on the ES scale (all three participant groups) showed a significant main effect of group (*F*=3.27, *P*=0.041), time (*F*=18.48, *P*<0.0001) and an interaction (*F*=9.89, *P*<0.0001). *Post hoc* tests found no significant group differences at any time point. In both patient groups, endocrine symptoms increased significantly between T1 and T2 (*t*=6.97, *P*=0.0001, *t*=3.83, *P*< 0.0001 chemotherapy and nonchemotherapy groups, respectively) and remained high at T3.

Quality of life measures did not correlate with individual test scores at baseline, T2 or T3. Quality of life scores were not significantly lower in those participants with reliable decline on multiple measures at T2 or T3.

#### HRT use and treatment-induced menopause

The potential effect of HRT use on cognitive function was examined across groups (the groups were balanced on the proportion of postmenopausal women who had taken HRT). There was no significant main effect of HRT use (never *vs* current or past) on any cognitive measure at baseline and HRT use was not associated with reliable decline on multiple measures at T2 or T3. Patients who experienced a treatment-induced menopause (TIM) following chemotherapy treatment (*N*=32 out of 39) were compared with those in the chemotherapy group who were postmenopausal at baseline. Those who had TIM were more likely to show reliable decline on multiple measures at T2 (OR=2.6, 95% confidence interval (CI) 0.823–8.266, *P*=0.086) and 1.51 times as likely at T3 (95% CI 0.405–3.922, *P*=0.145). These were not more likely to have above threshold GHQ_12_ scores at either time point or to report lower quality of life.

## DISCUSSION

This study benefits from both a longitudinal design and the ability to control for individual difference factors such as intelligence, age and education to permit a realistic appraisal of the extent of cognitive impairment after treatment for early stage breast cancer. Little convincing evidence was found to suggest that there is measurable and meaningful impairment for the vast majority of women in the UK who receive standard adjuvant treatments for breast cancer.

The most reliable predictor of performance on the tasks was age, intelligence and years in education. Chemotherapy, endocrine therapy, HRT use, quality of life and level of psychological distress were not associated with performance at a group or individual level. Although reliable decline on multiple measures was more common in the patient groups than in the healthy control group, there was not a significant association at either time point. More importantly, the majority of patients either showed no change or an improvement in performance. Many studies that classify patients as impaired or not, established on ‘failing’ a number of tests at a given criteria, are cross sectional and do not account for a change in cognitive function and other factors such as practice effects. Some of the issues relating to classifying cognitive impairment, using different statistical methods are detailed elsewhere (Shilling *et al*, 2005b).

A timely meta-analysis warns of the previous overinterpretation of apparent cognitive impairment in patients receiving chemotherapy. Although statistically significant cognitive impairment was found in the analysis, the magnitude of the impairment was small to moderate, with patients treated with chemotherapy performing better than the comparison group on some tests (Falleti *et al*, 2005). One possible reason why relatively little impairment was found in our chemotherapy group may be that the majority received relatively low dose FEC. This would support findings by van Dam *et al* (1998) who found no differences between patients receiving FEC (and also tamoxifen) and those not treated with systemic adjuvant therapy.

The level of psychological morbidity in the patient groups in our study was high at baseline but similar to that reported in the literature (Burgess *et al*, 2005). A difference between groups became apparent at the second assessment, with the chemotherapy patients maintaining high levels of psychological distress. The disparity between groups probably reflected the stage of treatment that the patients had reached. The nonchemotherapy patients had completed their radiotherapy treatment by several months, whereas many of the chemotherapy patients were waiting to begin. In common with other studies, psychological distress, quality of life measures and self-reported cognitive failures did not impact on objective tests of cognitive function, but were significantly associated with each other.

The hypothesis that women may experience greater cognitive decline if treatment results in a sudden premature menopause was supported, albeit weakly, probably due to the small sample size; these women were 2.6 times more likely to show reliable decline on multiple measures, following chemotherapy. A sudden menopause brings all the accompanying endocrine symptoms, for example hot flushes, night sweats and difficulty in sleeping. An accumulation of these may have interfered with a woman's ability to concentrate on the memory and attention tasks. The suggestion that an increase in individual endocrine symptoms may contribute significantly to cognitive performance was supported by the preliminary analysis of 50 of the present chemotherapy patients and 43 healthy controls (Shilling *et al*, 2005a). The most significant factor to account for the difference between the current data and that previously reported was that 52% of the patients in the preliminary analyses were premenopausal at baseline, compared to 36% in the present data set and these women were significantly more likely to experience a treatment-induced menopause (OR 3.12, *P*=0.016).

In summary, the results from this study suggest that only a small proportion of women receiving adjuvant treatments for breast cancer experience objective measurable change in their concentration and memory. It is reassuring that the majority are either unaffected or even improve over time, but such results are rarely emphasised in publication. The group of women who appear to be most at risk of exhibiting a decline in cognitive performance are those who experience a treatment-induced menopause, particularly in the initial period following chemotherapy. Future studies may like to focus on this group of women receiving treatments that induce early menopause, including LHRH therapy. This would also help clarify whether it is a reduction in oestrogen producing severe endocrine symptoms that interferes with attending to stimuli and therefore constrains memory processing, or chemotherapy treatment *per se*.

## Figures and Tables

**Table 1:** Age (at baseline), IQ, years in education and psychological distress

	**Chemotherapy (*N*=85)**	**Nonchemotherapy (*N*=43)**	**Control (*N*=49)**
Age (years)	51.49 (9.57)	58.93 (7.27)	51.90 (6.87)
IQ	109.89 (12.34)	111.05 (11.24)	112.04 (9.14)
Years in education	12.02 (2.60)	11.44 (1.76)	12.63 (2.86)
			
*Menopausal status*			
Premenopausal	39 (46%)	7 (16%)	13 (26%)
Peri/Postmenopausal	46 (54%)	36 (84%)	36 (73%)
			
*HRT use*			
Never	18 (39%)	13 (36%)	14 (39%)
Current			15 (42%)
Past	28 (61%)	23 (64%)	7 (19%)
			
*Above threshold GHQ_12_ scores* a ^,^ b			
T1	46(55%)	26(62%)	8(16%)
T2	43(51%)	10(24%)	9(18%)
T3	14(18%)	9 (21%)	12(24%)

a Data missing from 1 chemotherapy patient at T1 and T2 and from 7 at T3.

b Data missing from one nonchemotherapy patient at each time point.

**Table 2:** Treatment details for both patient groups

	**Chemotherapy group (*n*=85)**	**Nonchemotherapy group (*n*=43)**
*Tumour grade*		
Grade 1	4	16
Grade 3	31	25
Grade 3	50	2
		
*Type of surgery*		
WLE	47	36
Mastectomy	26	7
Mastectomy and reconstruction	7	1
Bilateral mastectomy	4	
Missing	1	
		
*Type of chemotherapy*		
FEC^a^ × 6 cycles	51	
FEC^b^ × 8 cycles	8	
CMF × 6 cycles	2	
4FEC 4 docetaxel × 8 cycles	3	
AC × 4 cycles	4	
EC × 6 cycles	9	
4EC 4 paclitaxel × 8 cycles	5	
4E 4CMF × 8 cycles	1	
4E 4FEC × 8 cycles	1	

a Data missing from one patient.

b Data missing from one patient.

**Table 3:** Results of regression analyses on baseline scores shows that combinations of education, age and Full Scale Intelligence Quotient (FSIQ) consistently predicted performance on the cognitive tasks

**TASK**	**R^2^**	**R^2^adj**	**F**	***P***	**Factor**	***β***	***t***	***P***
Letter cancellation	0.152	0.137	10.188	<0.0001	Education	0.489	1.889	0.061
					Age	−0.171	−2.734	0.007
					FSIQ	0.136	2.442	0.016
Auditory verbal learning task (AVLT)	0.109	0.98	10.444	<0.0001	Age	−0.049	−3.505	0.001
Supraspan					FSIQ	0.033	3.006	0.003
AVLT total	0.204	0.194	21.862	<0.0001	Age	−0.35	−5.319	<0.0001
					FSIQ	0.208	4.04	<0.0001
AVLT delayed	0.146	0.136	14.563	<0.0001	Education	0.304	0.085	<0.0001
					Age	−0.077	0.024	0.002
Complex figure immediate	0.112	0.101	10.689	<0.0001	Age	−0.209	−3.939	<0.0001
					FSIQ	0.104	2.509	0.013
Complex figure delayed	0.139	0.129	13.789	<0.0001	Age	−0.201	−4.169	<0.0001
					FSIQ	0.124	3.28	0.001
Story immediate recall	0.206	0.196	22.125	<0.0001	FSIQ	0.122	5.24	<0.0001
					Age	−0.125	−4.207	<0.0001
Story delayed recall	0.209	0.2	22.593	<0.0001	FSIQ	0.121	5.141	<0.0001
					Age	−0.134	−4.438	<0.0001
Letter/number sequencing	0.223	0.209	16.258	<0.0001	FSIQ	0.102	6.033	<0.0001
					Age	−0.075	−3.94	<0.0001
					Education	−0.179	−2.279	0.024
Digit span forward	0.212	0.207	46.278	<0.0001	FSIQ	0.094	6.803	<0.0001
Digit span backwards	0.221	0.212	24.205	<0.0001	FSIQ	0.086	6.593	<0.0001
					Age	−0.039	−0.2363	0.019
Spatial span forwards	0.118	0.108	11.453	<0.0001	Age	−0.053	−3.863	<0.0001
					FSIQ	0.031	2.906	0.004
Spatial span backwards	0.216	0.202	15.609	<0.0001	Education	0.15	2.663	0.008
					Age	−0.052	−3.77	<0.0001
					FSIQ	0.028	2.318	0.022
Stroop	0.084	0.079	15.722	<0.0001	Education	0.793	3.965	<0.0001

**Table 4:** Percentage of each group showing reliable decline, reliable improvement or no change for each task at T2 (T3)

	**Chemotherapy group (*n*=85)**			**Nonchemotherapy group (*n*=43)**			**Healthy control group (*n*=49)**		
	**Decline**	**Improve**	**Stable**	**Decline**	**Improve**	**Stable**	**Decline**	**Improve**	**Stable**
Letter^a^ cancellation	4 (6)	2 (2)	94 (92)	0 (9)	5 (5)	95 (86)	4 (2)	4 (4)	92 (94)
AVLT supraspan	15 (14)	10 (9)	75 (77)	9 (5)	9 (5)	82 (90)	8 (6)	16 (6)	76 (88)
AVLT total	12 (12)	4 (6)	84 (82)	9 (16)	5 (12)	86 (72)	6 (8)	4 (6)	90 (86)
AVLT delayed	5 (5)	2 (5)	93 (90)	2 (0)	12 (12)	86 (88)	6 (0)	4 (6)	90 (94)
Complex figure imm.^b^^,^^c^	5 (5)	8 (12)	87 (83)	12 (2)	5 (13)	84 (85)	4 (4)	2 (4)	94 (92)
Complex figure del.^d^	7 (1)	7 (12)	86 (87)	5 (2)	2 (2)	93 (96)	2 (0)	4 (6)	94 (94)
Story imm.	8 (11)	11 (12)	81 (77)	14 (7)	14 (14)	72 (79)	6 (8)	6 (4)	88 (88)
Story del.	10 (1)	15 (8)	75 (91)	9 (5)	9 (5)	82 (90)	2 (2)	4 (4)	94 (94)
Letter/number sequencing	5 (10)	3 (5)	92 (85)	14 (9)	0 (2)	86 (89)	6 (8)	8 (4)	86 (88)
Digit span forward	6 (4)	3 (2)	91 (94)	7 (2)	0 (5)	93 (93)	10 (4)	2 (4)	88 (92)
Digit span backwards	2 (5)	0 (3)	98 (92)	7 (5)	0 (9)	93 (86)	8 (2)	4 (2)	88 (96)
Spatial span forwards	0 (2)	3 (18)	97 (80)	0 (7)	5 (9)	95 (84)	10 (6)	4 (0)	86 (94)
Spatial span backwards	5 (4)	3 (1)	92 (95)	5 (0)	7 (2)	88 (98)	2 (6)	8 (2)	90 (92)
Stroop^e^^,^^f^	11 (4)	5 (8)	84 (88)	9 (7)	2 (9)	88 (84)	6 (4)	6 (4)	88 (92)

Missing data.

a Time 3; data missing from one chemotherapy patient and one nonchemotherapy patient.

b Time 2; data missing from one healthy control.

c Time 3; data missing from one chemotherapy patient and one healthy control.

d Time 3; data missing from one chemotherapy patient.

e Time 2; data missing from one chemotherapy patient and one healthy control.

f Time 3; data missing from two chemotherapy patients and one healthy control.

**Table 5:** Extent of reliable change at T2 and T3

	**T2**			**T3**		
	**Chemo**	**Nonchemo**	**Control**	**Chemo**	**Nonchemo**	**control**
Decline on ⩾2 measures	17 20%	11 25.6%	9 18.4%	15 18.1%	6 14.3%	5 10.6%
Improve on ⩾2 measures	19 22.4%	7 16.3%	8 16.3%	27 32.1%	13 30.2%	7 14.6%

## Appendix 1

The RCI was calculated as follows:

The test–retest reliability coefficient (*r*_xx_) was computed for each measure. The standard error of measurement (SE_m_) was calculated by:

where SD_1_ is the s.d. of the baseline score. The standard error of the difference (SE_diff_) was calculated by:

The standard error of the difference describes the spread of distribution of change scores that would be expected if no change occurred.

To establish a 90% RC confidence interval, the SE_diff_ was multiplied by ±1.64 s.d. (Kneebone *et al*, 1998). These cutoff points were corrected for practice effects (Sawrie *et al*, 1996). The practice effect for each variable is the mean difference between the follow-up and baseline scores. Thus, for each variable, an RC 90% confidence interval was calculated by:

For each participant, a difference score was calculated representing the performance difference on each measure (T2 – T1). If this score fell outside the RC interval, a statistically significant change in performance on this measure was considered to have occurred.
